# Polymer Derived Ceramics from Si, B, SiB_6_, and Mo_5_SiB_2_ Filler-Loaded Perhydropolysilazane Precursors as Protective and Functional Coatings for Refractory Metal Alloys

**DOI:** 10.3390/ma13214878

**Published:** 2020-10-30

**Authors:** Iryna Smokovych, Caren Gatzen, Manja Krüger, Michael Schwidder, Michael Scheffler

**Affiliations:** 1 Institute of Materials and Joining Technology, Otto-von-Guericke University Magdeburg, P.O. Box 4120, D-39016 Magdeburg, Germany; manja.krueger@ovgu.de (M.K.); m.scheffler@ovgu.de (M.S.); 2 Institute of Energy and Climate Research, Microstructure and Properties of Materials (IEK-2), Forschungszentrum Jülich GmbH, D-52428 Jülich, Germany; c.gatzen@fz-juelich.de; 3 Institute of Chemistry, Otto-von-Guericke University Magdeburg, P.O. Box 4120, D-39016 Magdeburg, Germany; michael.schwidder@ovgu.de

**Keywords:** polymer derived ceramics, preceramic polymers, perhydropolysilazane, oxidation barrier coatings

## Abstract

Oxidation barrier coatings on the base of a perhydropolysilazane precursor with different amounts of Si, B, SiB_6_ and Mo_5_SiB_2_ (T2) fillers for the oxidation protection of Mo-Si-B alloys were developed. The influence of different filler compositions as well as the influence of pyrolysis conditions (temperature and atmosphere) was studied. The coating slurries were examined with respect to their rheological behavior, which allows optimization of the coating slurry. Dilatometry studies show that the coefficient of thermal expansion of the composites can be matched to refractory, especially molybdenum alloy based, substrates by varying the content and the composition of the filler. The pyrolyzed coating systems offer a low porosity, which is one of the key parameters to a high oxidation protection capability.

## 1. Introduction

A process–cost–effective alternative to various coating techniques such as pack cementation coating, magnetron sputtering and plasma spraying is offered by coating processes with preceramic polymers (ceramic precursors) under ambient conditions [1]. After their thermal conversion (pyrolysis) into polymer-derived ceramics, they may serve as protective and functional layers for metallic materials [2,3]. In our previous works, oxidation barrier coatings were developed using a perhydropolysilazane precursor and homogeneously distributed in it Mo_5_SiB_2_, SiB_6_, Si and B powder fillers [4,5]. These coating systems are intended to protect Mo-base alloys, in particular Mo-Si-B, Mo-Hf-B and Mo-Zr-B alloys, against high temperature oxidation above 1100 °C. Mo-base alloys containing high volume fractions of the Mo solid solution phase are prone to an infinite oxidation in the temperature range from 400 to 800 °C because of MoO_3_ formation and its instant evaporation up to the entire alloy’s destruction [6,7,8]. The oxidation protection mechanism of polymer–to–ceramic converted, homogeneously filled coating layers consists in the formation of a high temperature stable SiO_2_/SiNO matrix, which, in a combination with glass-forming filler particles, protects the alloy surface at the initial stage of oxidation [4,5,6]. The presence of the Mo_5_SiB_2_ (T2) phase in Mo-Si-B alloys leads to the formation of a self-protective SiO_2_-B_2_O_3_ layer with high viscosity that serves upon further oxygen penetration into the metal matrix at the later stage of oxidation [6,9,10]. Thus, the protective coating systems were developed taking into account the physical–chemical compatibility of the coating layers to Mo-Si-B, Mo-Hf-B and Mo-Zr-B substrates. 

The developed coating systems demonstrate a parabolic oxidation kinetic at 800 °C and 1100 °C in air up to 100 h [4,5]. The coatings were applied to plain Mo-, Mo-Si-B, Mo-Hf-B and Mo-Zr-B alloys [4,6] and Ti-alloys [11,12] using a technologically less challenging dip coating process. The phase composition and microstructure of coating systems, their oxidation protective properties and the process of the polymer-to-ceramic transition are described in detail elsewhere [4,5].

The aim of this work is to investigate the physical–mechanical properties of Mo_5_SiB_2_, SiB_6_, Si and B filled coating systems on the base of free-standing bulk samples without any contact to a Mo based alloy. The thermal expansion coefficient, density and porosity, flexural strength of the developed systems were studied in terms of the significant properties of oxidation barrier coatings. Examinations of the rheological behavior of the coating slurries are important for the application of the dip coating process.

## 2. Materials and Methods 

The coating systems were prepared using a perhydropolysilazane preceramic polymer (PHPS NN 120-20, Clariant Advanced MaterialsGmbH, Sulzbach, Germany) and particulate fillers from commercial suppliers: Si (99.50%, Thermo Fisher (Kandel) GmbH, Kandel, Germany), B (95.00–97.00%, Chempur, Feinchemikalien und Forschungsbedarf GmbH, Karlsruhe, Germany), SiB_6_ (98.00%). The Mo_5_SiB_2_ phase (T2) was produced by a powder metallurgical processing route using elemental powders of Mo (99.95%, H.C. Starck GmbH, Munich, Germany), Si (99.60%) and B (98.00%,) in high-purity quality. The elemental powders were mechanically alloyed for 20 h at 200 rpm in a planetary ball mill (PM 400, Retsch GmbH, Haan, Germany) with further heat treatment at 1400 °C for 1 h in a tubular furnace (Losic 1500-90-600-1, HTM Reetz GmbH, Berlin, Germany, heating/cooling rate of 5 K/min). Subsequently, the annealed powder was grinded for 1 h at 200 rpm in a planetary ball mill to achieve a median particle size of ~5 µm. According to XRD analysis (PANalytical GmbH, Kassel, Cu Kα radiation, λ = 0.1541 nm), the resulting powder consisted mainly of Mo_5_SiB_2_ phase with some residual Mo (according to the Ritveld quantification method, 2.23%). Some properties of the filler powders are summarized in Table 1.

Preceramic slurries were mixed in a specific precursor/filler volume ratio and were subjected to rheological examinations. Schematic sample codes are given in Table 2. Rheological measurements of the coating slurries were conducted with a rotational viscometer MCR 301 (Anton Paar Germany GmbH, Ostfildern-Scharnhausen/Germany) in a plate-to-plate configuration with a gap size of 1 mm. The measurements were carried out at room temperature under variation of the shear rate from 10^−1^ s^−1^ to 10^−3^ s^−1^.

For thermal expansion measurements, flexural strength, porosity and density investigations bulk samples were manufactured by a manually manageable doctor blade (ZUA 2000, Zehntner GmbH, Sissach, Switzerland). The bulk samples of the size 25 mm × 5 mm × 2.5 mm were obtained by four times covering of the first layer. The samples were crosslinked in a drying furnace at 110 °C for 1.5 h, then subjected to pyrolysis in Ar or N_2_ atmospheres at 1000 °C or 1200 °C for 1 h; heating and cooling rates were 3 K/min, respectively. The dilatometric examinations were carried out using a horizontal dilatometer, model DIL 402 C (Netzsch, Selb, Germany), in the temperature range 25–1300 °C in Ar or N_2_ at 1000 °C or 1200 °C on pyrolyzed samples; the heating rate was 5 K/min in Ar. The thermal expansion coefficients were evaluated and calculated using the Proteus software (Netzsch, Selb, Germany).

To determine the density and porosity of the bulk samples, a helium pycnometer (AccuPyc 1330, Micromeritics GmbH, Aachen) was applied. Five measurements of each sample were conducted to evaluate the average values.

Three-point bending tests were carried out to determine the flexural strength of the bulk samples. Bar-shaped specimens of 25 mm × 2.5 mm × 2.5 mm (±0.2) were produced according to DIN EN 843-1. Testing was carried out with a universal testing machine (Zwick/Roell Z100, Zwick GmbH & Co. KG, Ulm, Germany). A pre-stress of 5 N was applied to the samples, and the test samples were subjected to a crosshead speed of 0.1 mm/min. The values of bending strengths, $\sigma_{f}$, were calculated in accordance with the Equation (1):

(1) $$\sigma_{f}=\frac{3\mathrm{Fl}}{2{\mathrm{bh}}^{2}},\;\mathrm{MPa}$$

where F is the breaking strength,  l is the distance between the centers of the support rollers, and b and h are the width and the height of the sample, respectively.

## 3. Results

### 3.1. Rheological Properties

An estimation of the rheological properties of the coating slurries is required for the formation of homogeneous coating layers in a dip coating or doctor blade process. The developed coating systems vary from each other in terms of filler volume fraction, particles size and physical properties of the starting filler powders (see Table 1). The uniformly dispersed condensed phases in the slurry are an important precondition to obtain thick coating layers from the filler containing precursor. This avoids sedimentation of the coating slurry and results in a coating thicknesses below the critical cracking point [20]. Another point is that filler containing systems should exhibit a shear thinning behavior due to low viscosity at high shear rates at the beginning of the coating procedure and high viscosity after the shear stress is ceased. 

In this study, a plate–plate measuring system was applied for the viscosity determination of the coating systems. According to this measuring system, the share rate values cohere with a withdrawal speed of the dip coating process [21]. Their correlation is presented in Equation (2):

(2) $${\dot{\gamma }}_{a}=\frac{2}{3}mm^{-1}\cdot U,\;\;s^{-1}$$

where ${\dot{\gamma }}_{a}$ is the average shear rate and U is the withdrawal speed. 

On the other hand, the withdrawal speed predetermines the thickness of the coating, $h_{0}$, in accordance with the Landau–Levich theory, Equation (3) [22]:

(3) $$h_{0}=c_{1}\sqrt{\frac{\eta \;U}{\rho \;g}},\;\mathrm{mm}$$

where $c_{1}$ is a proportionality constant, η is the viscosity of the slurry, ρ is the density of the slurry and g is the gravity constant. The Landau–Levich theory in the presented form is, however, limited to fluid systems with a Newtonian flow behavior.

Thus, the coating thickness is directly proportional to the withdrawal speed and the viscosity of the coating slurry, but inversely proportional to its density. 

**Figure 1:**
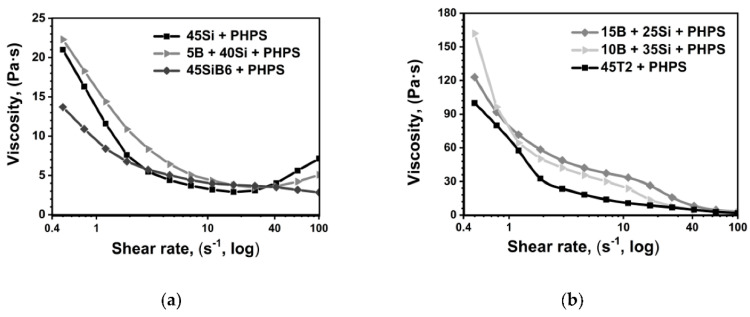
Viscosity curves of filler loaded coating slurries: (**a**) 45Si, 5B+40Si, 45SiB_6_ systems; (**b**) 15B+25Si, 10B+35Si, 45 T2 systems; measured at room temperature.

In Figure 1, the viscosity curves of the different coating systems are presented. The values of the viscosity of the 45Si, 5B+40Si and the 45SiB_6_ system are in the same order of magnitude (Figure 1a). Although the filler materials differ in particle size and particle shape, the densities of the Si, B and SiB6 powders are almost the same (see Table 1). Therefore, at low shear rates, the viscosity of the 45Si and the 5B+40Si systems with almost identical amounts of silicon fillers, 45 vol.% and 40 vol.%, respectively, is cognate.

At high shear rates (above 40 s^−^^1^), the 45Si and the 5B + 40Si systems demonstrate a dilatant effect—the shear thickening of the coating slurry by increased rates of a shear strain [23]. At these shear rates, the free movement of particles is significantly hampered, since collisions between fine powders increase. Dilatant is controlled by such factors as particle size, shape, crystal structure and distribution. Thus, a high amount of finely dispersed Si powders (4.45 μm) with a diamond cubic structure of a high compressive strength could lead to the shear thickening behavior of the 45Si and the 5B + 40Si coating slurries at high shear rates. In turn, the 45SiB6 system demonstrates a shear-thinning behavior due to the lower particle number of the relatively coarse SiB_6_ particles (18.99 μm) in the slurry. 

The effect of the particle concentration on the viscosity of the coating slurry is described by the Krieger–Dougherty equation [24]:

(4) $$\frac{\eta }{\eta_{m}}={\left(1-\frac{\varphi }{\varphi_{m}}\right)}^{-\left[\eta \right]\varphi_{m}}$$

where η is the viscosity of the slurry, $\eta_{m}$ is the viscosity of the base medium, φ is the solid particles concentration in the slurry, $\varphi_{m}$ is the maximum solid particle concentration in the slurry at which the flow can occur and [η] is the intrinsic viscosity of the medium.

This equation shows that, at low particle concentrations, the system possesses an almost Newtonian flow behavior. Increasing the particle concentration results in an increased particle–particle interaction, and as a result the resistance to flow increases. 

According to Equation (4), the higher viscosity values of 10B + 35Si and 15B + 25Si, with a high amount of fine B powder (2.32 μm) compared to 5B + 40Si and 45Si, were explained, see Figure 1b. The dilatant effect was not observed for these systems. Evidently, a rhombohedral crystal structure of boron is more exposed to deformations at high shear rates in comparison to the diamond cubic structure. It must be noted that similar rheological dates were obtained for the 50 vol.% filler loaded perhydropolysilazane precursor system in the work [25].

The viscosity of the 45T2 system appears to be the lowest within this slurry series because of the lower amount of T2 particles due to their high density (see Table 1). 

Taking into account the character of the viscosity curves of the coating systems considered in this section and the given Equations (2) and (3), it becomes clear that, when using different coating slurries, the thickness of the coating can be adjusted mainly by varying the withdrawal speed during the dip coating process; this process was applied in former studies [26].

### 3.2. Density and Porosity of the Coating Systems

**Figure 2:**
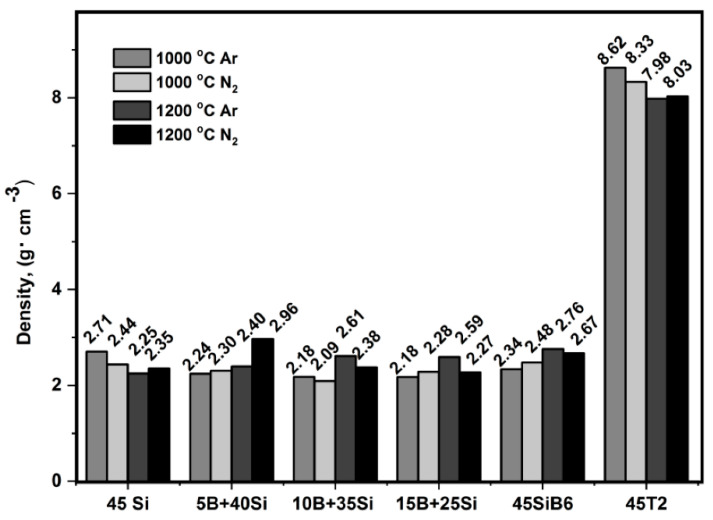
Skeletal density of bulk samples of the filler loaded coating systems after pyrolysis in Ar and in N_2_ at 1000 °C and 1200 °C.

Density and porosity are important physical and microstructure properties for environmental barrier coatings. The density of porous materials can be defined as a bulk density, ρ, which refers to the entire volume of the sample body, and this can be geometrically determined; the skeletal density, $\rho_{s}$, is the volume of the specimen without the volume of the open pores; the true density, $\rho_{0}$, refers to the volume of the solid material without all pores [25,27]. The skeletal density of bulk samples was measured using a helium pycnometer. The values of the skeletal density of bulk samples after pyrolysis in Ar or N_2_ at 1000 °C and 1200 °C are presented in Figure 2. To determine the true density, samples crushed prior to the measurement were investigated.

The density of boron and silicon loaded systems with different Si/B ratios, as well as the SiB_6_ system, vary among each other only to a minor extent after pyrolysis and independently of the pyrolysis conditions. Their densities range from 2.08 to 2.96 g·cm^−^^3^, which is related to the densities of the filler powders (see Table 1). The coating systems loaded with T2 phase have densities of 8.02—8.62 g·cm^−^^3^ that also correspond to the initial density of the pure T2 phase. The variation of the pyrolysis temperature (1000 °C or 1200 °C) and the pyrolysis atmosphere (Ar or N_2_) does not significantly affect the densities of the coating systems.

The open, Po, and closed, Pc, porosities were calculated using Equations (5) and (6):

(5) $$Po=\left(1-\frac{\rho }{\rho_{s}}\right)\times 100\;\%$$

(6) $$Pc=\left(1-\frac{\rho }{\rho_{0}}\right)\times 100\;\%-Po$$

**Figure 3:**
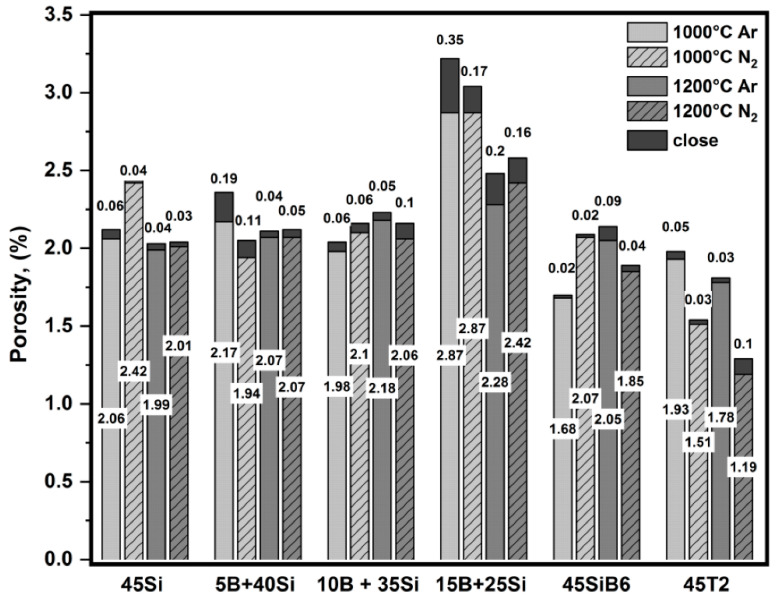
Open and closed porosity of samples after pyrolysis in Ar or in N_2_ at 1000 °C or 1200 °C; dark part of the bar: closed porosity.

Figure 3 presents the values of the open and closed porosity of the samples after pyrolysis in Ar and N_2_ at 1000 °C and 1200 °C.

The total porosity of the coating systems is low: the closed porosities do not exceed 0.4% after pyrolysis at 1000 °C and 0.2% after pyrolysis at 1200 °C; the open porosity is 2.9% at 1000 °C and 2.4% at 1200 °C. The high open porosity affects the resistive performance of the coating against oxidation, reducing its ability to protect against oxygen penetration, while the low closed porosity influences the effective elastic modulus, increasing the strain compliance of the coating [1]. The coating system (15B + 25Si) possesses the highest porosity due to the presence of a high amount of small B powder, see Table 1. An increase in the pyrolysis temperature leads to a decrease in porosity of the samples. 

### 3.3. Coefficient of Thermal Expansions

**Figure 4:**
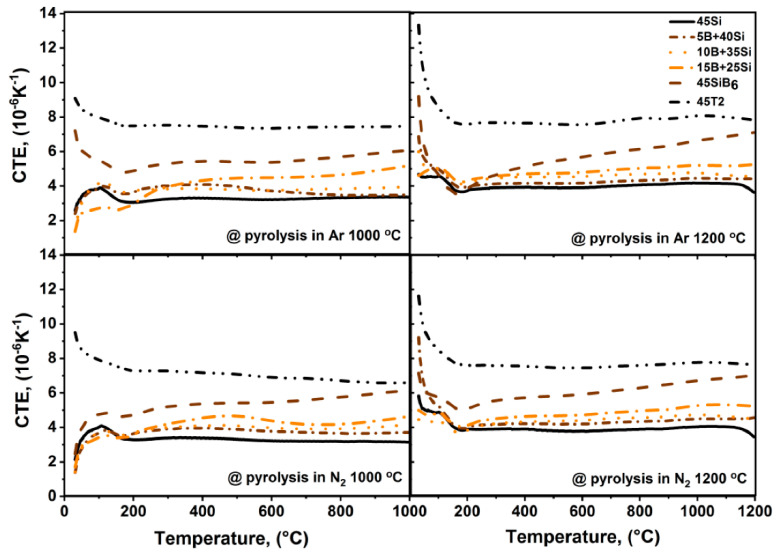
CTE values of bulk samples of the filler loaded coating systems after pyrolysis in Ar and in N_2_ at 1000 °C and 1200 °C.

Another important point in case of coatings is the thermal expansion behavior, thus the linear coefficient of thermal expansion (CTE) of bulk samples was measured (Figure 4). Prior to the measurement of the thermal expansion up to 1200 °C in Ar, the samples were subjected to pyrolysis in Ar or N_2_ at 1000 °C or 1200 °C for 1 h. 

These results open the feasibility to evaluate the CTE mismatch between the coatings and a potential substrate. If the thermal behavior of the coating and the substrate are significantly different, thermally induced mechanical stresses occur, which increase the risk of coating spallation [2,28]. 

The CTE of most Mo-base alloys is in the range of 8–10 × 10^−6^ K^−1^, depending on the specific alloy’s phase composition. Thus, for the Mo_5_Si_3_ phase (T1) with the tetragonal crystal structure, the CTE of the a axis is ~5.7 × 10^−6^ K^−1^, while the CTE of the c axis amounts to 14 × 10^−6^ K^−1^ [29]. The CTE value of the MoSi_2_ phase along the a axis is 8 × 10^−6^ K^−1^ and along the c axis is 10 × 10^−6^ K^−1^ [29]; the CTE of the Mo solid solution phase varies from 5.35 to 6.5 × 10^−6^ K^−1^ [30], while for the T2 phase the CTE is 7.5–7.7 × 10^−6^ K^−1^ at room temperature [31]. 

According to the CTE measurements, the CTE of the 45T2 system is in the range of 7.3–7.5 × 10^−6^ K^−1^, independent of the pyrolysis conditions. This coincides well with the CTE value of T2 powder given in Table 1. For the 45SiB_6_ systems, the CTE varies from 5.2 to 6 × 10^−6^ K^−1^, which is also comparable to the initial CTE value of SiB_6_ powder. However, an increase in the CTE of up to 7.5 × 10^−6^ K^−1^ was observed with the increase in the temperature from 600 °C to 1200 °C. This may be connected to changes in the microstructure of the samples and the presence of large-scale defects such as cracks or pores; no reference for such thermal behavior of SiB_6_ was found for this temperature range.

The CTE of the 45Si system is 3–4 ×10^−6^ K^−1^, which is slightly lower than that of the Si/B systems 5B + 40Si, 10B + 35Si and 15B + 25Si. An increase in the boron content from 5 vol.% to 15 vol.% led to an increase in the CTE from 3.65 × 10^−6^ K^−1^ to 4.57 × 10^−6^ K^−1^. Thus, a reduction in the CTE mismatch between the coating and the Mo-base substrate might be carried out by an increase in the B-fraction in this system. 

It must be noted that the CTE of the polymer-derived SiCNO ceramic is 3–4 × 10^−6^ K^−1^ [2,32,33]. The coefficient of thermal expansion of the filler loaded PHPS is basically a combination of the CTE for pure (pyrolyzed) PHPS and the CTEs of the pure filler particles. In case no other phases are formed and the filler particles are homogenously distributed within the matrix, the CTEs follow the rule of mixture [34,35]. Therefore, the theoretical CTE values were calculated according to the Voigt approximation [34,35]:

(7) $$\alpha =\;\alpha_{1}+\left(\alpha_{2}-\alpha_{1}\right)\cdot V_{2}+\;\left(\alpha_{3}-\alpha_{1}\right)\cdot V_{3}$$

where $\alpha_{1}$, $\alpha_{2}$ and $\alpha_{3}$ are the CTEs for pure (pyrolyzed) PHPS and the filler phases, respectively, and $V_{2}$ and $V_{3}$ are the volume fractions of the corresponding filler phases. The theoretical CTE values are given in Table 3.

The measured CTE values of the 5B + 40Si, 10B + 35Si, 15B + 25Si and the 45SiB_6_ systems coincide well with the theoretical calculated ones. Some deviations may indicate reactions of the filler particles with the surrounding matrix. A significant deviation of the measured CTE of the 45T2 system from the calculated value might be caused by the presence of residual phases with higher CTEs in the starting T2 powder [9]. Nevertheless, the use of compatible substrate phases such as T2, T1 and other molybdenum silicides appears to be the most efficient method to reduce the CTE mismatch between the coating system and a potential, e.g., refractory alloy, substrate material.

### 3.4. Flexural Strength

**Figure 5:**
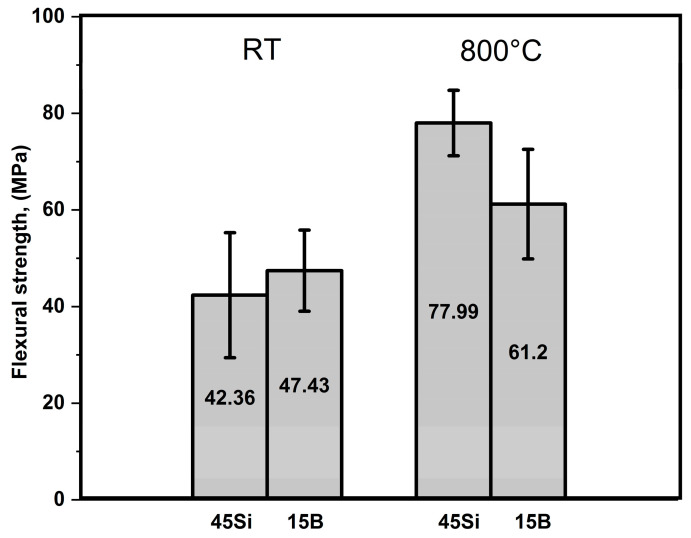
Flexural strength of 45Si and 15B + 25Si samples at room temperature (RT) and 800 °C in air.

Due to their high sensitivity to tensile stresses, the strength of the ceramic materials was determined by a bending test. Three-point bending was selected as a test setup. The experiments were carried out with pyloysed samples (1000 °C, 1 h in N_2_). The strength investigations were conducted at room temperatures in air and at 800 °C in Ar with a purity of 4.8. Since the high-temperature testing machine chamber provides three-point bending equipment only, measurements at room temperature were carried out also in this mode. The results of the bending tests are presented in Figure 5.

The average bending strength of 45Si and 15B + 25Si samples at room temperature are 42.36 MPa and 47.43 MPa, respectively. The slightly higher bending strength for 15B + 25Si, with higher content of fine powders than in 45Si, is initially surprising, but the difference lies in the range of the standard deviation. At 800 °C, an increase in the bending strength of the coating systems is observed: 77.99 MPa for 45Si and 61.2 MPa for 15B + 25Si. This may be connected to the formation of SiO_2_ or B_2_O_3_-SiO_2_ glassy scales on the surface, which cannot be completely avoided by performing the bending tests in a technically pure Ar atmosphere. The strengthening of the 45Si took place due to a steady Si oxidation under the condition of residual O_2_ penetration from the high-temperature measuring chamber and growing of the amorphous SiO_2_ layer. A gradually increased oxidation curve was observed for these samples during the cyclic oxidation test in air at 800 °C [5]. Evidently, this may explain some higher values of the flexural strength of 45Si and 15B + 25Si samples at room temperature (RT) and 800 °C in air. However, more detailed investigations of the influence of the glassy scale on the mechanical behavior of the coating systems are the subject of ongoing studies.

## 4. Conclusions

In this study, new preceramic polymer based coating systems with different amounts of Si, B, SiB6 and T2 fillers for the oxidation protection of refractory alloys were investigated. Since the viscosity is one of the key parameters during dip-coating processing, the relationships between the withdrawal speed of dip coating and the share rate values of the plate–plate measuring system were determined. By varying the withdrawal speed, the thickness of the coating can be adjusted.

Subsequently, further essential parameters for coatings were investigated on bulk samples of coating systems. The samples had a very low porosity regardless of the filler composition and pyrolysis conditions: the total porosity was from 1.3% to 3.3% and should not significantly affect the oxidation resistance. The coating densities correlated with the density of the starting filler powders. The use of MoSi_3_, T1 or T2 phases as powder fillers can be efficient in reducing the CTE mismatch between the coating and Mo-Si-B, Mo-Hf-B or Mo-Zr-B substrates. An increase in the boron content in the Si/B-filled systems enhances the CTE of the coatings and, in turn, may decrease the mismatch between the layer and a matrix.

In addition, three point bending tests were carried out at room temperature in air and at 800 °C in Ar with selected samples. The flexural strength seems to increase at 800 °C as compared to room temperature, which was attributed to the formation of SiO_2_ or B_2_O_3_-SiO_2_ glassy coating layers. However, more effort is necessary to further investigate the strengthening effect of the glassy scales on coating systems. 

The results in this study point out that viscosity during coating processing, porosity and coefficient of thermal expansion, as essential parameters and properties, can be tailored; the optimized coating systems based on preceramic polymers may be provided as oxidation barrier coatings to refractory alloys.

## Figures and Tables

**Table 1:** Some properties of filler powders.

Fillers	Average Particles Size, (μm)	Type of Crystal Structure	Density, (g/cm^3^)	Melting Point, (°C)	Coefficient of Thermal Expansion, (×10^−6^/K)
Si	4.45	Diamond cubic	2.57 [13]	1414 [13]	2.6 [14]
B	2.32	Rhombohedral	2.08 [15]	2076 [15]	5.0–7.0 [15]
SiB_6_	18.99	Orthorhombic	2.43 [16]	1864 [17]	6.0 [18]
Mo_5_SiB_2_ (T2)	4.73	Tetragonal	8.86 [19]	2120 [19]	7.55 [19]

**Table 2:** List of compositions and sample codes.

Sample Code	Filler Composition (Numbers Correspond to Vol.%)
45Si	45Si + 55 PHPS
5B + 40Si	5B + 40Si + 55 PHPS
10B + 35Si	10B + 35Si +55 PHPS
15B + 25Si	15B + 25Si +60 PHPS
45SiB_6_	45SiB_6_ + 55 PHPS
45T2	45T2 + 55 PHPS

**Table 3:** Theoretical and measured CTE values of the coating systems.

Sample	45Si	5B + 45Si	10B + 35Si	15B + 25Si	45 SiB_6_	45T2
Theoretical CTE (×10^−6^ K^−1^)	3.37	3.59	3.81	4.1	4.9	5.755
Measured CTE (×10^−6^ K^−1^)	3–4	3.65	4.09	4.57	5.2	7.3–7.5
